# Evaluation of Prescription Practices for Antenatal Steroids in Pregnant Women

**DOI:** 10.1007/s10995-025-04070-1

**Published:** 2025-02-20

**Authors:** Antalya Jano, Caroline Madigan, Paris Ekeke

**Affiliations:** University of Pittsburgh School of Medicine Pittsburgh, Pittsburgh, PA USA

**Keywords:** Disparity, Steroids, Community need, Respiratory, Preterm

## Abstract

**Objective:**

The significant racial disparity in adverse birth outcomes is unexplained by individual-level stressors. This implores us to explore modifiable prenatal care delivery characteristics. Our objective was to evaluate if racial disparities in infant respiratory outcomes were explained by inequitable exposure to antenatal steroids.

**Methods:**

We included women who delivered infants between 23 and 34 weeks gestation in Level 3 NICU between January 2017 and December 2020. Prenatal and postnatal variables, including exposure to antenatal steroids, were collected. A community need index was assigned designating level of neighborhood deprivation. Chi squared and *t* tests were performed to look for racial differences in prenatal and delivery characteristics.

**Results:**

There were no racial differences in steroid administration with 89.9% of eligible Black women and 89.8% of eligible White women completing an antenatal steroid course. Despite no differences in maternal risk factors such as diabetes, pre-eclampsia, prenatal care utilization and PPROM, Black infants were more likely to require intubation (p = 0.04), oxygen (p = 0.001), and surfactant (p = 0.008) in the delivery room compared to White infants. Compared to the lower community need groups exposed to ANS, the high need group had higher rates of chorioamnionitis and were more likely to be on Medicaid and Black race. Despite this, there were no differences in infant respiratory outcomes by community need group.

**Conclusions:**

Despite the shift in focus to include the interaction between individuals and their community exposures, the racial disparity in birth outcomes persists. Attention should be paid to other modifiable elements of a mother’s prenatal experience.

## Significance

There is a vast body of research examining adverse birth outcomes which note a persistent racial disparity where Black women have more preterm births and low birth infants compared to White women. Previous studies have focused on individual level risk factors such as maternal age, prenatal care utilization, other medical comorbidities, and socioeconomic status, but none of these factors singularly account for the racial disparity. Our study attempts to incorporate variables on a community level (area deprivation) and system level (antenatal steroid exposure) to account for racial differences in the respiratory outcomes of infants.

## Introduction

Infants born to Black women have consistently poorer perinatal outcomes than those born to White women; neonatal death and stillbirth is twice as likely in Black infants than in white infants (Steer, 2005). While Black infants are at increased risk of being born preterm or small for gestational age, there is no doubt that the inequities Black pregnant women face contribute to some degree to the differential rates of adverse perinatal outcomes (Ford & Airhihenbuwa, 2010; Mendez et al., 2014; Sheikh et al., 2022). These inequities are sharply reflected, and often even more pronounced on the local level. In Pittsburgh, PA, the city in which our study’s patients received their maternal–fetal care, Black women are three times more likely to give birth to extremely low weight infants and the Black maternal mortality rate is higher than corresponding mortality rates in 97% of similar cities despite the fact that Pittsburgh’s Black women start prenatal care earlier and have lower rates of gestational diabetes, hypertension, and infection (Howell et al., 2019). The racial disparities seem to also correlate with geographic disparities and neighborhood demographics (Howell et al., 2019).

These alarming statistics beg the question of the interplay between systemic racism, neighborhood deprivation, and other environmental maternal stressors that may directly influence poor fetal outcomes. Historically, we have been unsuccessful at explaining the etiology of perinatal racial disparities using individual level risk factors, thus more recent studies have focused on examining the cumulative effects of risk factors at the societal, community, and individual levels (Gadson et al., 2017; Masho et al., 2014; Wallace et al., 2013). However, even after accounting for maternal age, socioeconomic status, and medical comorbidities, a racial disparity persists (Masho et al., 2014). This trend implores us to closely examine other contextual factors such as exposure to environmental and social stressors such as area deprivation. Area deprivation is considered a marker of socioeconomic disadvantage. Indices of area deprivation can describe small areas such as neighborhoods or municipalities or larger areas such as census tracts or counties, but often incorporate assessments of income, employment, financial status, and housing quality to make a multi-faceted assessment (Messer et al., 2006; O’Campo et al., 2008).

Several studies have suggested that deprivation at the neighborhood level is significantly associated with increased risk of preterm birth among both non-Hispanic White women and non-Hispanic Black women (Messer et al., 2006; O’Campo et al., 2008; Ma et al., 2015). When considering racial disparities in birth outcomes, area deprivation may serve as an indirect measure of systemic racism including redlining, exposure to violence, and decreased access to health-promoting behaviors due to proximity to health clinics, fresh produce, and walking trails. Because the primary driver of poor infant outcomes is prematurity, many public health initiatives focus on optimizing maternal health to reduce preterm birth and other adverse birth outcomes. The American College of Obstetricians and Gynecologists (ACOG) has established general standards of prenatal care to minimize the risk of prematurity and its complications. Per ACOG guidelines, antenatal steroid (ANS) administration should be considered for pregnant women at risk for preterm delivery at the limits of viability until 34 weeks (Committee on Obstetric Practice, 2017). Steroids after 34 weeks is more controversial (Andrikopoulou et al., 2021). Research indicates that this practice is strongly associated with decreased neonatal morbidity and mortality, as ANS are known to promote fetal lung maturity (Roberts et al., 2017). If the parental goals of care are in line with life prolongation, a course of corticosteroids is recommended for those within 7 days of anticipated preterm delivery less than 34 weeks and has been shown to reduce neonatal rates of respiratory distress syndrome (RDS), intraventricular hemorrhage (IVH), necrotizing enterocolitis, need for mechanical ventilation (Roberts et al., 2017). Despite the evidence for antenatal steroids, the ambiguity in risk stratification for preterm delivery has led to variable administration rates across the US; for instance, previous studies suggesting that pregnant Black women were less likely to receive ANS than pregnant White women (Gulersen et al., 2020; Ondusko et al., 2022). In light of these findings, there is an implication that some of the racial disparity in birth outcomes stems from inequitable hospital practices and biased care.

## Objectives

Due to the large body of existing literature studying individual level risk factors as predictors of poorer Black infant outcomes, we sought to incorporate health system delivery and geographic factors, such as antenatal steroid use and area deprivation to further explain racial disparities in infant respiratory outcomes. The primary aim was to ascertain if the racial disparity in infant respiratory outcomes could be explained by inequitable exposure to antenatal steroids. We hypothesized that of all women eligible for antenatal steroids, Black women may be offered less than White women.

## Methods

We conducted a retrospective cohort study approved by the Institutional Review Board of the University of Pittsburgh. All women who delivered a liveborn, singleton infant between gestational age of 23 0/7 weeks and 33 6/7 weeks at Magee Women’s Hospital in Pittsburgh, PA from January 1, 2017 to December 31, 2020 were included. They were deemed eligible to receive antenatal steroids if they were considered to be at risk for imminent delivery within 7 days per ACOG guidelines. Data was limited to this time period to minimize confounding due to changes in prenatal care administration due to COVID and practice changes regarding resuscitation at the limits of viability. Exclusion criteria consisted of multiple gestations, presence of congenital anomalies, and GA less than 23 0/7 or more than 33 6/7 weeks. Participants were also excluded if they elected comfort measures only for their infant (no resuscitation) or if data on pertinent risk factors were incomplete.

Data was extracted from the Vermont Oxford Network (VON), an electronic database of infant variables from all deliveries at Magee Women’s Hospital, a level 3 NICU in Pittsburgh, Pennsylvania. For each participant, maternal and infant clinical variables were collected. The following maternal variables were collected: presence of adequate prenatal care, antenatal steroid exposure including number of doses; history of prior preterm birth, preterm premature rupture of membranes (PPROM), mode of delivery, maternal age, diabetes mellitus, hypertension, pre-eclampsia, and chorioamnionitis; maternal insurance and relationship status. Adequate prenatal care was defined as absence of diagnosis of late, limited, or no prenatal care, thus women had to have established care in first trimester and attend at least 80% of prenatal visits (Martin & Osterman, 2023). Infant variables including gestational age, birth weight, and respiratory requirements in the delivery room and during hospitalization were also noted. Subsequently, we examined the difference in antenatal steroid exposure stratified by race. Then we examined racial differences in short- and long-term respiratory outcomes in infants stratified by antenatal steroid exposure. Short term outcomes include a diagnosis of respiratory distress syndrome, need for intubation, oxygen, and surfactant in the delivery room. Long term respiratory outcomes include diagnosis of bronchopulmonary dysplasia (BPD) or need for oxygen at 36 weeks corrected gestational age. Pearson’s chi-squared tests were used for categorical variables and t-test used for continuous variables.

The residential address for each participant at time of delivery was geocoded into their corresponding municipalities or neighborhoods. Based on a participant’s municipality, a Community Need Index (CNI) was assigned to one of 5 groups: Very Low Need, Low Need, Moderate Need, High Need, Extreme Need. The Community Need Index was developed by the Allegheny County Department of Human Services (DHS) to identify communities that are in greater need relative to others. The index uses indicators such as poverty, unemployment, and gun violence to identify the census tracts that have the highest levels of need. The community need index was only applicable to those who resided in Allegheny County so those with patients who resided outside of the county were excluded from parts of the sub analysis. Subsequent sub analysis examined differences in delivery characteristics stratified by community need group.

## Results

**Fig. 1:**
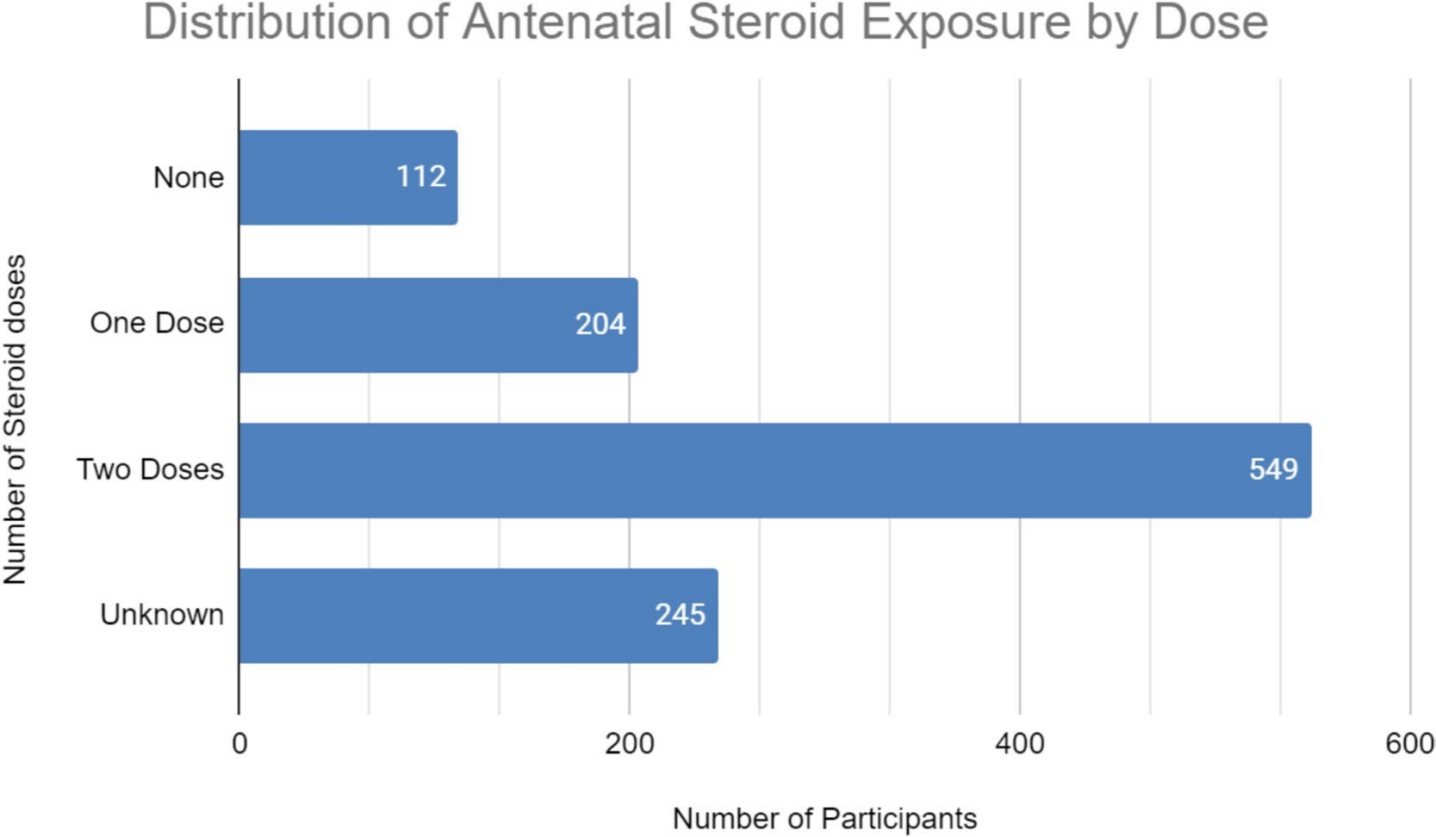
Distribution of Antenatal Steroid Exposure by Dose

There was a total of 1110 women identified to be eligible for antenatal steroids in the period of interest. Of the eligible women, the racial distribution included 64.8% (709/1110) white women, 26.8% (298/1110) Black, 3.3% (37/1110) Asian, 1.1% (12/1110) denoted “other race”, and 4.6% (51/1110) Unknown race. Figure 1 outlines the distribution of those who received antenatal steroids with 10.1% of eligible women receiving no antenatal steroids, 18.4% receiving one dose of antenatal steroid, and 49.5% received a full course (2 doses of antenatal steroids 24 h apart). The remaining 22.1% were denoted unknown initially but when able were reclassified based on manual chart review (Table 1).

**Table 1:** Number of Antenatal Steroid Dose by Race

	Black N = 298	White N = 709
No Steroid Doses	31(10.4%)	66(9.3%)
1 Dose of Steroid	59(19.8%)	123(17.3%)
2 Doses of Steroid	156 (52.3%)	355(50.1%)
Unknown Number of Doses	52(17.5%)	165(23.3%)

Due to very small numbers of other races, the subsequent analysis focused on differences between Black and white women leaving us with a cohort of 1,007 women. Baseline characteristics in our cohort are depicted in Table 2. Black women had slightly younger infants (avg gestational age 29.5 weeks vs 30.4 weeks, p < 0.001) and higher rates of chorioamnionitis (14.7% vs. 10.4%, p = 0.04) compared to White women. There were no racial differences in antenatal steroid administration with 89.9% of eligible Black women and 89.8% of eligible White women completing at least first dose of antenatal steroid course. There were no statistically significant differences in prenatal care utilization, maternal diabetes, maternal hypertension, pre-eclampsia, PPROM, and Cesarean section rates. When comparing differences in infant respiratory outcomes stratified by race, Black infants were more likely to require intubation (p = 0.04), oxygen (p = 0.001), and surfactant (p = 0.008) in the delivery room compared to White infants (Table 3). Of those that received antenatal steroids, we found there was no significant racial difference in timing of administration of antenatal steroids prior to delivery (Table 4).

**Table 2:** Baseline Characteristics of Perinatal Factors in Eligible Women

Variable	Black (N = 298)		White (N = 709)		P value
	n	%	n	%	
ANS exposure^1^	268	89.9	637	89.8	0.97
Average Gestational Age (wks)	29.5		30.4		< *0.001*
Maternal Age	27.9		29.7		< *0.001*
Chorioamnionitis	44	14.7	74	10.4	*0.04*
History of Prior Preterm Birth	72	24.2	161	22.7	0.647
Maternal Diabetes Mellitus (Gestational or Chronic)	43	14.4	150	21.2	0.08
Maternal Hypertension (Gestational or Chronic)	104	34.9	251	35.4	0.87
Pre-eclampsia	75	25.2	208	29.4	0.31
PPROM^2^	217	72.8	494	70.0	0.34
Prenatal Care^3^	281	94.3	677	95.5	0.71
Method of Delivery					0.48
Vaginal	122	40.9	251	35.4	
Cesarean section	176	59.1	458	64.6	

1 Antenatal Steroid exposure (Minimum of 1 dose of corticosteroid prior to delivery)

2 Preterm Premature Rupture of Membranes defined as rupture of membranes prior to 37 weeks

3 Presence of Adequate Prenatal Care

**Table 3:** Infant Respiratory Outcomes by Race

Variable	Black (N = 298)		White (N = 709)		P value
	n	%	n	%	
Neonatal Bronchopulmonary Dysplasia (BPD)	38	12.9	89	12.6	0.76
Intubation in Delivery Room	72	24.2	131	18.4	*0.04*
Surfactant in Delivery Room	46	15.4	68	9.6	*0.008*
Oxygen in Delivery Room	232	77.9	476	67.1%	*0.001*
Neonatal Mortality	16	5.4	22	3.1%	0.085

**Table 4:** Timing of Antenatal Steroid Administration Prior to Delivery by Race

	Black N = 268	White N = 637
< 24 h	59(22%)	124(19.5%)
> 24 h	113(42.1%)	265(41.6%)
> 7 days	68(25.4%)	160(25.1%)
Unknown Timing*	28(10.4%)	88(13.8%)

^*^ANS documented as being given by birth hospital but timing unclear

Lastly, we examined we grouped those that were exposed to antenatal steroids by community need group with the high need group defined as those women who resided in an area classified by the Community Need Index as “Extreme” or “High” need and compared their delivery characteristics to women who resided in areas classified as “Moderate” or “Low” need (Table 5). Due to this Community Need Index being used to compare areas within Allegheny County exclusively, we excluded all eligible women who lived outside of Allegheny County in our sub analysis, resulting in a final cohort of 469/905(51.8%) women who received ANS and resided in Allegheny County.

**Table 5:** Differences in Delivery Characteristics by Community Need for Women Exposed to Antenatal Steroids

Variable	Extreme-High Need N = 184 (n, %)	Very Low-Moderate Need N = 285 (n, %)	P value
Mean Birth Weight (grams)	1455	1514	0.134
Avg GA (weeks)	30.1 (SD 2.77)	30.4(SD 2.76)	0.343
Maternal age (years)	28.8 (SD 6.38)	30.5(SD 5.43)	0.053
No Prenatal Care	6 (3.3)	8(2.8)	0.69
Insurance Type			< *0.001*
Medicaid	148 (80.4)	134 (47.0)	
Private	36 (19.6)	151 (53.0)	
Maternal Race			< *0.001*
Black	111 (60.3)	85 (29.8)	
White	59 (32.1)	169 (59.3)	
Other	14(7.6)	31(10.9)	
Maternal Hypertension	70(38.0)	97(34.0)	0.376
Maternal DM	19 (10.3)	23 (8.1)	0.277
Pre-eclampsia	51 (27.7)	82 (28.8)	0.787
Chorioamnionitis	25 (13.6)	22 (7.7)	*0.039*
CPAP in DR	147 (79.9)	222 (77.9)	0.593
ETT in DR	43 (23.3)	65 (22.8)	0.821
O2 in DR	143 (77.7)	218 (76.5)	0.758
Surfactant during Stay	46 (25.0)	83 (29.1)	0.329
Ventilation	60 (32.6)	121 (42.5)	0.580
Mean NICU Stay (d)	39.2 (SD 2.92)	41.7 (SD 2.74)	0.682
RDS	100 (54.3)	162 (56.8)	0.302

Of these 469 women that were prescribed antenatal steroids, 60.8% were classified as low moderate need, while 39.2% were classified as high/extreme need. There were no significant differences in gestational age by level of community need. However, those in high need neighborhoods had slightly lower birth weights compared to those in low need areas (1455 g vs. 1514 g, p = 0.134), though these values were not statistically significant. 60.3% of black women in Allegheny County were classified as high or extreme need, while 32.1% of white women in Allegheny County were classified as high or extreme need, p < 0.001. Women from high community need areas were more likely to be on Medicaid compared to women from low community need areas (80.4% vs. 47.0%, p < 0.001). There was no statistically significant difference in any maternal risk factors, including maternal age, hypertension, diabetes, or pre-eclampsia. There was a higher rate of chorioamnionitis in the high community group compared to the low community need group (13.6% vs 7.7%, p = 0.039). When stratified by community need group, there was no difference in rates of ETT, CPAP, surfactant or oxygen administration in the delivery room.

## Conclusions

Despite no significant difference in antenatal steroid exposure, Black infants had poorer respiratory outcomes compared to White infants. They were more likely to require resuscitation in the delivery room with higher rates of oxygen, intubation, and need for surfactant. There were no statistically significant racial differences in bronchopulmonary dysplasia (BPD), length of stay or mortality prior to discharge which suggests that the disparity is not primarily mediated by the quality of in-hospital care in the NICU. One possible explanation is that there are additional stressors in the prenatal environment that affect how well Black infants fare in the immediate postnatal period. Stressors such food or housing instability, interpersonal conflict, chronic exposure to discrimination and psychological stress contribute to the increased allostatic load and higher baseline cortisol levels in people from disadvantaged and minority backgrounds (Vianna et al., 2011; Barrett et al., 2018; Feinberg et al., 2016). There is a growing body of research that shows exposure to prenatal stress is associated with alterations in pro-inflammatory cytokine levels (Coussons-Read et al., 2005; Ekeke et al., 2020; Wright et al., 2010). This may also explain why chorioamnionitis was more prevalent in Black infants via the inflammatory pathway. It is plausible that repetitive exposure to prenatal stressors can activate one’s fight or flight response and activate the inflammatory pathway disrupting Black women and their infant’s homeostasis, but it is likely many of these stressors occur outside of the hospital setting.

Area deprivation indices such as the community need index attempt to capture some of the social stressors we experience in our daily lives that alter our metabolic milieu and may have short- and long-term effects on an individual’s health outcomes. The fact that there were more Black women represented in the high need group compared to White women could explain some of the disparity in infant respiratory outcomes, but our study was not powered to truly quantify this potential effect. Despite this, it is plausible that exposure to high community need areas in isolation does not make a statistically appreciable effect on perinatal outcomes but in conjunction with medical comorbidities and the chronic stress of lifelong exposure to microaggressions, overt and covert racism, and other elements of social disadvantage there is a cumulative negative impact on the health of Black women and their infants.

Although this study further highlights the disparities in outcomes for Black infants, it also demonstrates the difficulty in examining maternal care as a continuum from prenatal maternal variables to postnatal short and long-term infant outcomes. Prior studies on traditional medical risk factors fail to account for persistent racial disparities in birth outcomes. Research including environmental risk factors such as neighborhood deprivation also have shown to be associated with adverse birth outcomes but when taken singularly do not fully account for the pervasive racial disparity. Previous studies have implicated the difference in quality of prenatal maternal medical care by race, but the results of our study also seem to suggest that the racial disparity in infant outcomes is not explained by traditional medical risk factors even when considering neighborhood deprivation and suggests there are out of hospital factors not being captured that likely play a role. Further conclusions are limited due to this study being performed retrospectively and the imprecision in data collection from the electronic medical record. Another limitation of the study was the Vermont Oxford Database is mostly reflective of infants born at the level III neonatal intensive care unit (NICU), thus mortality and long-term respiratory outcomes likely were underestimated due to the proportion of babies that were transferred to higher level of care. Infants are not accounted for in the VON database until they are discharged from their initial hospitalization so those infants that have more severe respiratory disease that are transferred to our level 4 NICU have delayed entry into VON. In general, the number of total infants who need tracheostomies is low in our institution (< 10%) so is unlikely to significantly affect the distribution, but admittedly we do not have access to the data of transfers to the level 4 NICU by race so that is a limitation. Information about intrauterine fetal demise (IUFD) is limited and thus these infants were excluded from our study since there would be no data in Vermont Oxford Network to pull from, thus there is potential for a survival bias in our cohort.

Unfortunately, community level data as a proxy for environmental stress is limited. Much is still unknown about racial disparities in maternal and infant health. Although there was no appreciable difference in antenatal steroid exposure, Black infants fared worse in the delivery room compared to White infants. Further studies are needed to elucidate the cumulative effect of social determinants of health more carefully at the community level and how these community exposures directly or indirectly affect a specific individual’s outcome.

## Data Availability

Data pulled from Vermont Oxford Network database with center specific results that are not accessible to the public without specific permissions.

## Code availability

STATA codes can be made available if needed.
