# Correction: Evaluation of Prescription Practices for Antenatal Steroids in Pregnant

**DOI:** 10.1007/s10995-025-04088-5

**Published:** 2025-03-27

**Authors:** Antalya Jano, Caroline Madigan, Paris Ekeke

**Affiliations:** University of Pittsburgh School of Medicine Pittsburgh, Pittsburgh, PA USA

**Correction to: Maternal and Child Health Journal**

10.1007/s10995-025-04070-1

The original version of this article contained an error in percentages of chorioamnionitis under Results section. The value should have been 14.7% vs. 10.4% instead of 10.3% vs. 6.8%.

Both incorrect and correct versions of the sentence under Results section is presented here.

**Incorrect:**

Black women had slightly younger infants (avg gestational age 29.5 weeks vs. 30.4 weeks, *p* < 0.001) and higher rates of chorioamnionitis (10.3% vs. 6.8%, *p* = 0.04) compared to White women.

**Correct:**

Black women had slightly younger infants (avg gestational age 29.5 weeks vs. 30.4 weeks, *p* < 0.001) and higher rates of chorioamnionitis (14.7% vs. 10.4%, *p* = 0.04) compared to White women.

The original article has been corrected.

